# JNK–NQO1 axis drives TAp73-mediated tumor suppression upon oxidative and proteasomal stress

**DOI:** 10.1038/cddis.2014.408

**Published:** 2014-10-23

**Authors:** A Kostecka, A Sznarkowska, K Meller, P Acedo, Y Shi, H A Mohammad Sakil, A Kawiak, M Lion, A Królicka, M Wilhelm, A Inga, J Zawacka-Pankau

**Affiliations:** 1 Department of Biotechnology, Intercollegiate Faculty of Biotechnology, University of Gdansk and Medical University of Gdansk, Gdansk, Poland; 2 Department of Microbiology, Tumor and Cell Biology, Karolinska Institutet, Stockholm, Sweden; 3 Centre for Integrative Biology, CIBIO, University of Trento, Mattarello, Italy

## Abstract

Hyperproliferating cancer cells produce energy mainly from aerobic glycolysis, which results in elevated ROS levels. Thus aggressive tumors often possess enhanced anti-oxidant capacity that impedes many current anti-cancer therapies. Additionally, in ROS-compromised cancer cells ubiquitin proteasome system (UPS) is often deregulated for timely removal of oxidized proteins, thus enabling cell survival. Taken that UPS maintains the turnover of factors controlling cell cycle and apoptosis – such as p53 or p73, it represents a promising target for pharmaceutical intervention. Enhancing oxidative insult in already ROS-compromised cancer cells appears as an attractive anti-tumor scenario. TAp73 is a *bona fide* tumor suppressor that drives the chemosensitivity of some cancers to cisplatin or *γ*-radiation. It is an important drug target in tumors where p53 is lost or mutated. Here we discovered a novel synergistic mechanism leading to potent p73 activation and cancer cell death by oxidative stress and inhibition of 20S proteasomes. Using a small-molecule inhibitor of 20S proteasome and ROS-inducer – withaferin A (WA), we found that WA-induced ROS activates JNK kinase and stabilizes phase II anti-oxidant response effector NF-E2-related transcription factor (NRF2). This results in activation of Nrf2 target – NQO1 (NADPH quinone oxidoreductase), and TAp73 protein stabilization. The observed effect was ablated by the ROS scavenger – NAC. Concurrently, stress-activated JNK phosphorylates TAp73 at multiple serine and threonine residues, which is crucial to ablate TAp73/MDM2 complex and to promote TAp73 transcriptional function and induction of robust apoptosis. Taken together our data demonstrate that ROS insult in combination with the inhibition of 20S proteasome and TAp73 activation endows synthetic lethality in cancer cells. Thus, our results may enable the establishment of a novel pharmacological strategy to exploit the enhanced sensitivity of tumors to elevated ROS and proteasomal stress to kill advanced tumors by pharmacological activation of TAp73 using molecules like WA.

Targeted therapeutics such as kinase inhibitors have emerged as promising drugs for cancer patients but as for the chemotherapy regimes, drug resistance, often linked to oncogenic reprogramming of cancer cells, confines the effectiveness of these therapies.^1, 2, 3^

Redox homeostasis is essential to sustain cellular functions and enable cell survival. In normal cells, modest reactive oxygen species (ROS) modulates many cellular processes through stress-kinase pathways, including mitogen-activated protein kinase (MAPK) pathway.^4^ Interestingly, ROS is emerging as master regulators of embryonic stem cells' renewal, proliferation, immune response and differentiation.^5, 6, 7, 8^ Increased oxidative stress burden, often linked to the chronic inflammation, is implicated in the tumorigenesis and leads to the elevated anti-oxidative response program in stress-adapted tumors. As a result, it often confers chemoresistance to the ROS-driven treatments.^9, 10, 11, 12, 13^ Based on this knowledge, a new therapeutic concept is being developed that focuses on exploiting master regulators of the anti-oxidant response, such as NRF2, to overcome the tumor resistance.^11^

In normal cells, elevated levels of ROS often promote an enhanced activity of the proteasome system to remove the oxidized proteins, thus to prevent their toxicity. This phenomenon is observed in many human neurodegenerative diseases, including Alzheimer's disease, Parkinson's disease and Huntington's disease.^14, 15, 16, 17, 18, 19^ Similarly, ROS-compromised cancer cells, for timely removal of oxidized proteins, possess an enhanced system for the protein ubiqutination and an amplified activity of the ubiquitin proteasome pathway (UPP), in particular 20S proteasome particle.^14^ The 20S proteasome particle is additionally important for the degradation of the liable tumor suppressors like p53 and TAp73, the process rescued by NADPH quinone oxidoreductase (NQO1).^20^ With the approval of bortezomib, to treat multiple myeloma, inhibition of the proteasome activity emerges as a promising anti-cancer therapy.^21^

Withaferin A (WA) and withanone (WN) are steroidal lactones obtained from *Withania sommnifera* – medical herb of Ayurveda.^22^ They possess anti-inflammatory and anti-cancer properties. WA inhibits catalytic β-subunit of 20S proteasome core particle similarly to the clinically used proteasome inhibitor (PI) bortezomib^23, 24, 25, 26^ and was shown to impede NF-*κ*B function in cancer cells^27^ via stabilization of I*κ*B*α*, the cytoplasmic inhibitor of NF-*κ*B. Similarly, withanolide D inhibited degradation of I*κ*B*α* by targeting the UPP.^28^ Anti-tumor functions of WA were also linked to its direct interactions with annexin II,^29^ anti-angiogenic activity,^28^ inhibition of STAT-1/3 and Akt signaling pathways and subsequent stabilization of p53 by activated ARF, which inactivates MDM2 and prevents its binding to p53.^30^ Taken together, WA is a potent inhibitor of tumorigenesis both *in vitro* and *in vivo*, but the exact mechanism underlying WA anti-cancer activity remains unresolved.

Pharmacological recapitulation of p53 activity is currently exploited as a new therapeutic strategy to combat cancers.^31^ Several p53 reactivating small molecules have been developed and are currently undergoing preclinical or clinical developments.^31,32^ Their implementation in clinics, mainly due to the adverse pharmacokinetics, is still impeded. This, together with the fact that p53 is lost or mutated in >50% of all human cancers, indicates that novel approaches are needed for the effective tumor killing.

*TP73* belongs to the p53 family of genes, including *TP63* and *TP53*. The role of p63 and p73 in cancer is determined by the existence of two classes of isoforms, TA (transcriptionally active) and ΔN (transcriptionally inactive N terminus deleted). TA isoforms structurally resemble p53 and act as tumor suppressors.^33^ p53, TAp63 and TAp73 can transactivate many of the same target genes and execute their tumor-suppressor function by guarding the genomic stability and promoting cell cycle arrest, replicative senescence or apoptosis^34, 35, 36^ Around 70% of *TAp73*^−/−^ mice develop tumors (mostly lung adenocarcinomas), and the rest show premature aging linked to the deregulated metabolism.^37^ This indicates that TAp73 is a tumor suppressor and important regulator of metabolism. Unlike *TP53*, which is mutated in about 50–80% of human cancers, the *TP73* gene mutations are rare.^34^ Therefore TA isoforms of p73 can compensate for p53 function in tumors with lost or mutated p53.

In cancer cells, the transcriptional activity of TAp73 is kept in check by several protein inhibitors, such as ΔNp73, ΔNp63, iASPP^38^ and aurora kinase A,^39^ and mutant p53 form heterocomplexes with TAp73 and abrogate its transcriptional activity.^33,34,38, 39, 40^ MDM2, E3 ubiquitin ligase that binds p53 and promotes its ubiquitin-dependent degradation, blocks TAp73 transcriptional activity via direct binding to its transactivation domain but does not promote TAp73 proteolytic disassembly. Similar to p53, TAp73 can be directed for degradation in an ubiquitin-independent manner, which is mediated by 20S proteasomes.^20^ Asher *et al.*^20^ reported that NQO1 protein directly interacts with TAp73*α* via its SAM domain, and protects it from the proteasomal cleavage.^20^ IR-mediated DNA damage and oncogenic insult both activate TAp73 by releasing it from its negative regulators, such as MDM2, MDMX or iASPP.^34,38^ We and others have shown that TAp73 can serve as a therapeutically relevant target of anti-cancer molecules, including Nutlin,^41^ 37AA peptide,^42^ RETRA^43^ and protoporphyrin IX.^44^ This implies the ‘druggable' nature of TAp73 protein however, solid reports supporting the prominent, tumor-suppressive outcome of TAp73 restoration are still missing.

Selivanova and colleagues^45^ have previously shown that small-molecule RITA, a known p53 activator, promotes p53-mediated cell death in cancer cells by synthetic lethal mechanism converging on concurrent inhibition of thioredoxin reductase (TrxR), which results in elevated oxidative stress and inhibition of p53/MDM2 complex. This amends p53 from growth suppressor to effective apoptosis inducer.^46^ In the present study, we discovered that ROS insult is indispensable for an efficient induction of apoptosis by TAp73 upon treatment with proteasomal inhibitor – WA. This is a new direction in the field of pharmacological modulation of p73 pathway for efficient tumor killing that can be further exploited to develop potent anti-cancer agents, such as WA.

## Results

### Cell death is detected in tumor cells with *TP*53 gene deletion but not in normal cells after WA treatment

**Figure 1:**
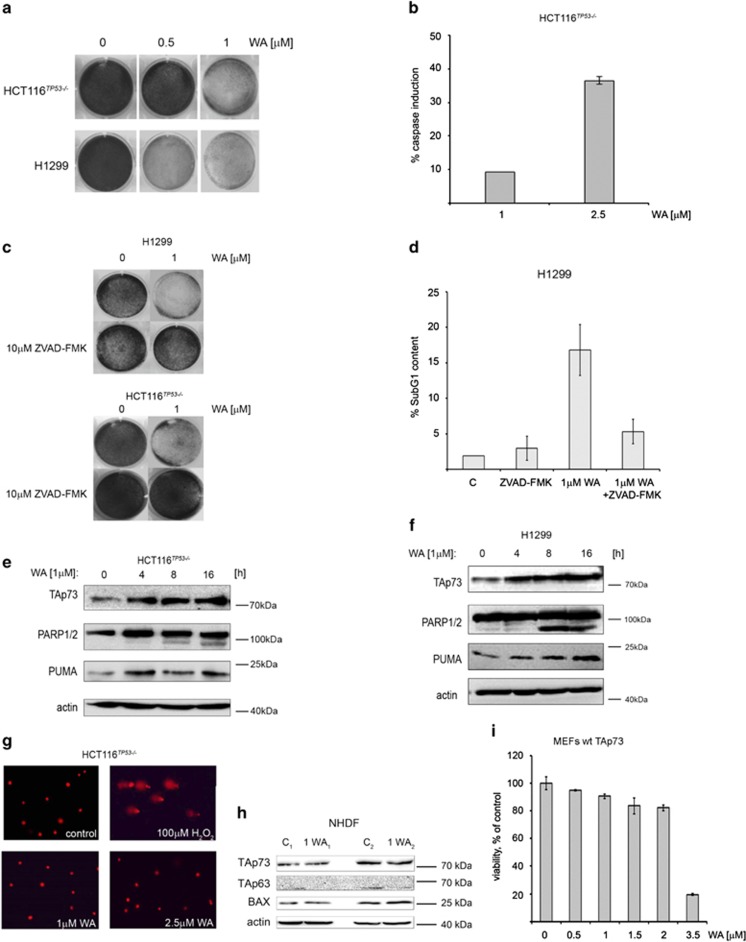
WA promotes apoptosis in cancer cells lacking p53. (**a**) H1299 and HCT116^*TP53*−/−^ cells were treated with WA for 48 h and stained with crystal violet for clonogenic survival determination. (**b**) Growth inhibition correlated with caspase activation by WA. (**c**) Caspase inhibitor zVAD-FMK protects H1299 and HCT116^*TP53*−/−^ from growth inhibition by WA. (**d**) WA stimulates accumulation of H1299 in subG1 fraction, which is reversed by zVAD-FMK pretreatment. (**e** and **f**) Immunoblots presenting induction of TAp73 in HCT116^*TP53*−/−^ and in H1299 cells, which coincides with the induction of apoptotic PUMA and with the PARP cleavage. (**g**) HCT116^*TP53*−/−^ cells after treatment with WA or H_2_O_2_ (100 *μ*M) for 3 h ( × 100 magnification) (*n*=3). (**h**) Immunoblot of WA-treated NHDF presenting TAp73, TAp63 and BAX protein levels. (**i**) WST-1 assay performed in MEFs wt *TAp73* treated with WA

WA (for structure, refer to Supplementary Figure S1A) was shown to exert anti-tumor activity in cells expressing mutant or wild-type p53.^29,47^ To address the question whether cancer cells lacking p53 but expressing TA isoform of p73 are dying upon treatment with WA, we performed viability and clonogenic assays in H1299, human lung adenocarcinoma cells and in HCT 116^*TP*53−/−^ human colon cancer cell line previously engineered for homozygous deletion of *TP53* gene resulting in p53 protein loss of function.^48^ As the median inhibition concentration of 50% (IC_50_) was 0.79 *μ*M in H1299 (48 h) as compared with 3.9 *μ*M in fibroblasts (normal human dermal fibroblasts (NHDF)) (Supplementary Figure S1B), we applied 0.5 and 1 *μ*M WA in our studies. Clonogenic survival assay revealed significant decrease in cell number already at low doses of WA in H1299 cells (Figure 1a, Supplementary Figure S6A), and 1 *μ*M was effective in both cell lines tested. This correlated with the increased levels of active caspases (Figure 1b), while the use of the pan-caspase inhibitor zVAD-FMK partially reversed WA-mediated inhibition of cell proliferation (Figure 1c, Supplementary Figure S6B). Consistent with caspase activation, WA induced SubG1 cell population in treated cells, which regressed after zVAD, a pan caspase inhibitor, pretreatment (Figure 1d).

**Figure 2:**
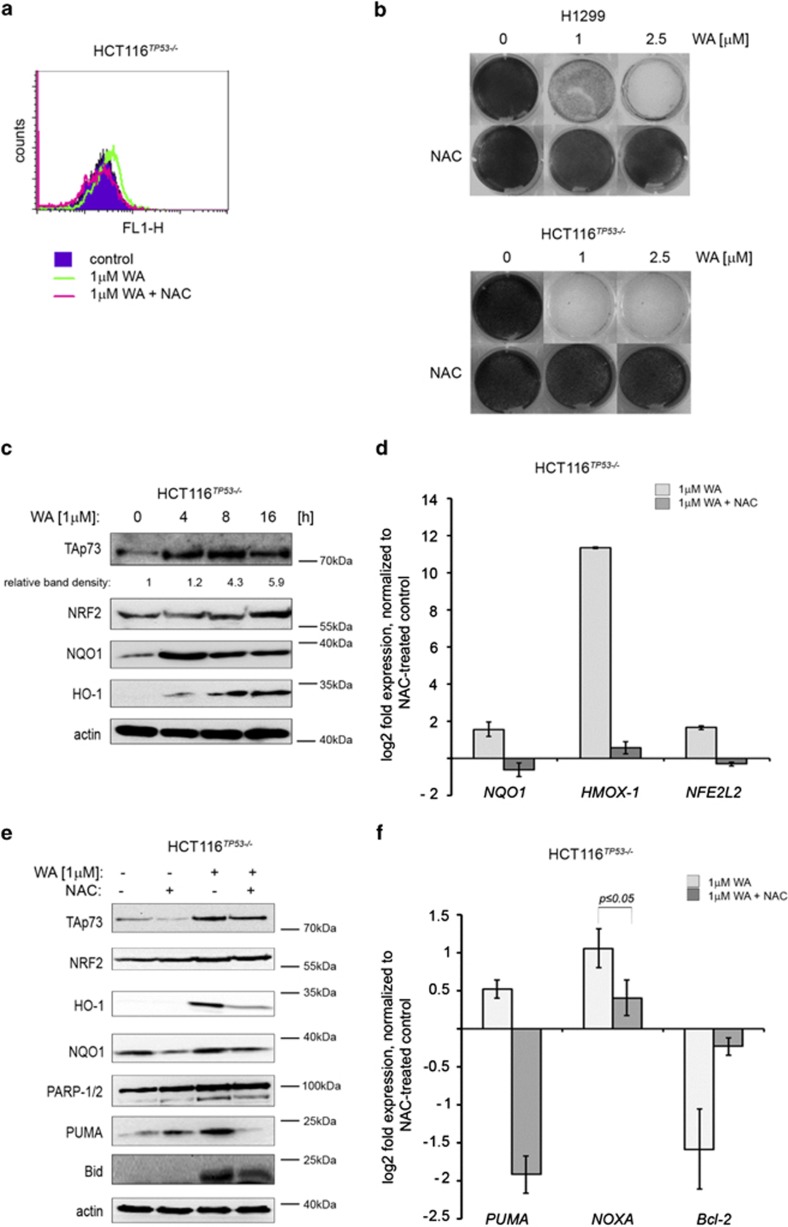
WA induces ROS and anti-oxidant response in tumor cells to trigger cell death. (**a**) DCF-DA-stained H1299 cells show elevated ROS levels upon WA treatment, which was prevented by NAC pretreatment. (**b**) ROS scavenger NAC abrogates WA-induced growth suppression in H1299 and HCT116^*TP53*−/−^ cells. (**c**) WA-induced ROS upregulates protein levels of TAp73 and PUMA along with NRF2 and its anti-oxidant effectors NQO1 and HO-1. (**d**) ROS induced the expression of anti-oxidant response genes *NFE2L2*, *NQO*1 and *HMOX-1* whereas NAC pretreatment reversed this effect. (**e**) Immunoblots of WA-treated HCT116^*TP53*−/−^ cells show induced TAp73 protein levels, which correlated with the induction of NRF2, NQO1, HO-1, PUMA, Bid and PARP. This effect was prevented by NAC pretreatment for 8 h that is also apparent from (**f**) qPCR analysis

Upon stresses such as DNA damage or oncogene activation, TAp73, similarly to p53, regulates transcription of apoptotic genes, including *PUMA*, *NOXA* and *BAX*.^34^ We found that WA promoted stabilization of TAp73 at the protein level, which correlated with the induction of cleaved PARP p85 fragment indicating that cells were dying of apoptosis (Figures 1e and f). Western blotting analysis revealed that the increase in TAp73 protein levels in cancer cells correlates with the elevated protein levels of PUMA, Bax and Bid. qPCR analysis shows that mRNA levels of *TAp73* and *ΔNp73* were not significantly affected upon WA (Supplementary Figures S1C and D), while we observed upregulation of TAp73 pro-apoptotic target *PUMA* and *NOXA* and downregulation of *Bcl-2* (Figure 2f).

As the systemic toxicity of the existing anti-cancer drugs limits their broad application, we assessed the genotoxicity of WA in cancer cells and in NHDF. The use of human normal cells in toxicity studies is of key relevance when planning future clinical trials for new anti-cancer drugs. Alkaline comet assay implies that WA did not promote DNA-damage (Figure 1g, Supplementary Figure S1E), did not affect viability of NHDF at concentrations tested (Supplementary Figure S1B) and did not induce TAp73 and pro-apoptotic proteins in normal cells (Figure 1h). What is more, we could not detect toxicity of WA toward primary mouse embryonic fibroblasts (MEFs) at the concentrations tested (Figure 1i).

Taken together, we conclude that WA is a non-genotoxic agent that promotes cell death in tumor cells, which coincides with the increase of TAp73 protein levels, accumulation of apoptotic proteins PUMA and NOXA and cleaved PARP.

### WA induces oxidative stress, which facilitates TAp73 activation and inhibits proliferation of cancer cells

Hahm *et al.*^47^ described that WA inhibits oxidative phosphorylation, which is a consequence of the generation of superoxide due to mitochondrial dysfunction. To address the question whether the elevation of ROS by WA is cell line specific or not, we measured the generation of hydroxyl, peroxyl and other ROS by employing cell-permeable reagent 2′,7′-dichlorofluorescein diacetate (DCF-DA) and measuring its fluorescence upon activation. Our experiments provide the evidence that WA effectively induced ROS in cancer cell lines deprived of p53 (Figure 2a, Supplementary Figure S2A). Pretreatment of cells with ROS scavenger *N*-acetyl-L-cysteine (NAC) inhibited accumulation of ROS by WA and ablated the anti-proliferative effect of WA in H1299 and HCT 116^*TP*53−/−^ cells (Figures 2a and b and Supplementary Figure S2A, and S6C and D).

In cells, oxidative stress activates effector proteins belonging to the anti-oxidant system response, including the master regulator – NRF2. Thus, next we investigated the downstream effectors of ROS triggered by WA and how they contribute to cancer cell death. As ROS triggers the anti-oxidant response, we sought to investigate whether WA activates NRF2 as a consequence of the induced oxidative stress.^49^ In our settings, we found that WA only slightly induced mRNA levels of NRF2 and efficiently promoted NRF2 stabilization on the protein level. It correlated with a potent overexpression of NRF2 target genes *NQO1* and heme oxygenase 1 (*HMOX-1* or HO-1), resulting in significant activation of proteins involved in phase II anti-oxidant response in HCT 116^*TP53*−/−^ and H1299 cells, which was reverted by NAC pretreatment (Figures 2c–e,Supplementary Figures S2A–C). This strongly implies that WA induces oxidative stress in cancer cells.

We reasoned that ROS induced by WA might promote TAp73 protein stabilization as NQO1, which expression depends on the oxidative status of the cell, was found to have a key role in ablating the Ub (ubiquitin)-independent degradation of p53 and TAp73.^20^ Indeed, pretreatment with NAC prevented the induction of TAp73 and PUMA proteins by WA (Figure 2e) and accordingly ablated the expression of TAp73 target genes *PUMA* and *NOXA* (Figure 2f). Furthermore, 1 *μ*M WA did not induce ROS in NHDF, which provides the explanation why normal cells are not affected by low dose of WA (Supplementary Figure S2D).

Thus WA promoted NRF2-related phase II anti-oxidant response as a consequence of induced oxidative stress. Next, our data indicate that oxidative stress induced by WA is critical for TAp73 stabilization and expression of its apoptotic targets *PUMA* and *NOXA*.

### TAp73 is essential for potent killing of cancer cells lacking p53 upon WA treatment

As ROS is an important activator of TAp73, we strived to investigate how WA-induced ROS activates TAp73 in cancer cells and whether TAp73 contributes to cell killing facilitated by WA.

**Figure 3:**
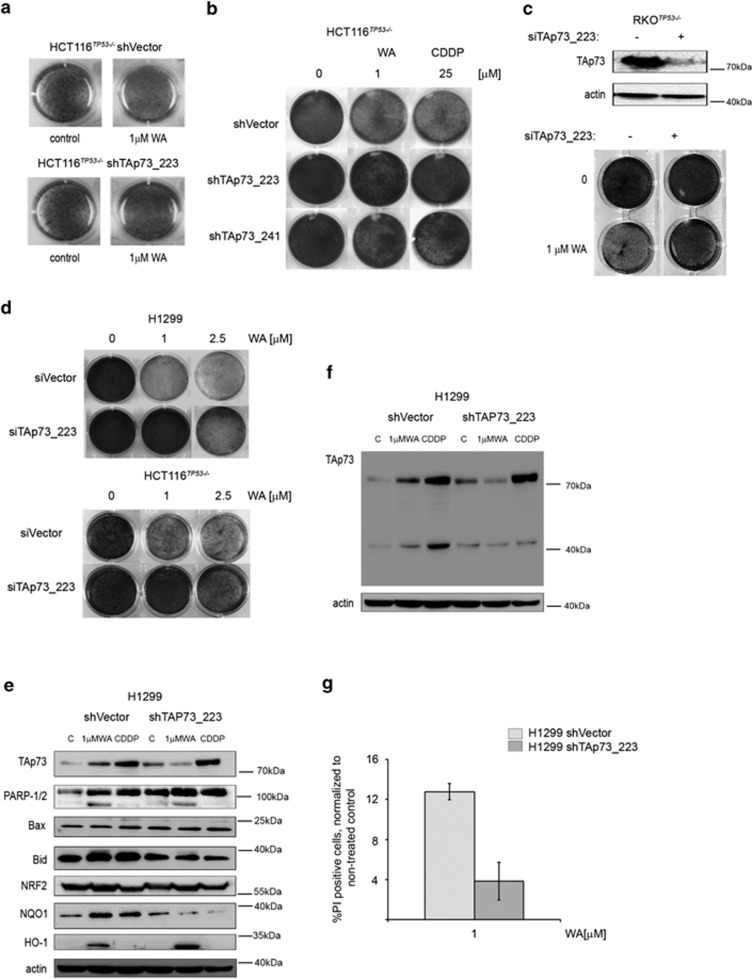
TAp73 directly contributes to cell killing by WA. (**a**) Soft agar assay performed in HCT116^*TP53*−/−^ stably knockdown for TAp73 cells and shVector transduced counterpart, shows reduced potential of these cells to form colonies upon WA treatment only when TAp73 is expressed. This is representative data of 3 independent experiments. (**b**–**d**) Stable and transient silencing of TAp73 ablates anti-proliferative function of WA in cancer cells as revealed by clonogenic assays. (**e**) Depletion of TAp73 in HCT116^*TP53*−/−^ cells prevents induction of the pro-apoptotic PUMA by WA. The shRNA control and shTAp73 samples were analyzed on the same membrane and exposed at the same time. (**f**) Whole membrane representing the levels of TAp73 upon shRNA-mediated knockdown indicates ineffective induction of TAp73 after WA treatment. (**g**) Propidium iodide staining of viable HCT116^*TP53*−/−^ shTAp73_223 shows reduced sensitivity to WA upon TAp73 depletion

To explore the role of TAp73 in WA-mediated cell death, we used both transient and stable TAp73 knockdown cell lines for further studies (Supplementary Figure S3B). Silencing of TAp73 expression, using two different shRNAs, led to the significant protection from WA-induced growth inhibition (Figures 3a–d, Supplementary Figures S3C and D and S7A). To elucidate whether TAp73 contributes to potent activation of apoptotic effectors by WA, we performed western blotting analysis in shVector- and shTAp73-expressing cells after WA exposure. We found that TAp73 depletion significantly reverted the induction of PARP cleavage, Bax and Bid accumulation by WA (Figures 3e and f), which implies that TAp73 is required for the promotion of apoptotic program upon WA. Additionally, this was further confirmed by the less prominent accumulation of propidium iodide-stained cancer cells' knockdown for TAp73 and treated with WA (Figure 3g).

### Phosphorylation of TAp73 by ROS-activated c-Jun N-terminal kinase (JNK) potentiates cell death triggered by WA

**Figure 4:**
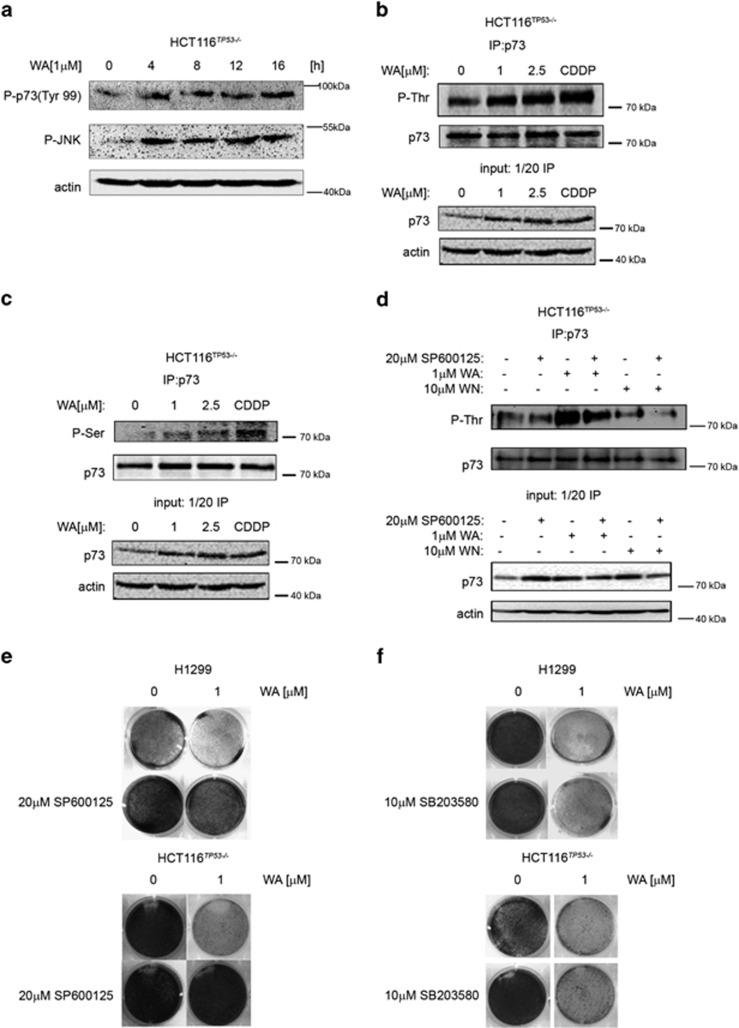
ROS-activated JNK kinase phosphorylates TAp73 and contributes to cell killing by WA. (**a**) WA triggers the phosphorylation of JNK kinase and TAp73. (**b** and **c**) WA promotes phosphorylation of threonine and serine residues in TAp73. (**d**) The SP600125 JNK inhibitor prevents phosphorylation of TAp73 on threonine residues by WA. (**e**) 20 *μ*M SP600125 significantly protects from WA-mediated growth inhibition. (**f**) SB203580, a p38 kinase inhibitor, does not protect H1299 and HCT116^*TP53*−/−^ cells from WA-mediated growth inhibition

It has been described previously that genotoxic stress induced by cisplatin activates c-Abl kinase, which in turn phosphorylates TAp73 at Tyr-99 leading to protein stabilization and activation.^50^ Therefore, in most of our studies we applied cisplatin (CDDP) as a positive control. The MAPK cascade includes p38 MAPK and JNK/SAPK (stress-activated protein kinase), which are important signal transduction messengers that are relevant for the regulation of cell motility, transcription and apoptosis. p38 and JNK/SAPK were shown to phosphorylate and activate TAp73 upon cellular stresses,^51^ which makes them candidate mediators of WA-induced oxidative stress to TAp73. Thus we found that WA promoted JNK and p38 kinases phosphorylation, which correlated with the phosphorylation of Tyr-99 in TAp73 (Figure 4a, Supplementary Figures S4A and B). TAp73-pTyr99 is a mark of DNA damage response and is executed by c-Abl kinase.^52^ As no DNA damage by 1 *μ*M WA was detected (Figure 1g), we speculate that the induction of pTyr99 might be a consequence of mitochondrial oxidative stress-mediated activation of DDR and is rather a downstream effect of the on-going apoptosis but not the DNA damage stress.^53^

The JNK kinase recognizes and phosphorylates serine and threonine residues, we thus performed phospho-specific IP for TAp73, which showed robust phosphorylation of p73 at multiple Ser and Thr residues upon WA treatment (Figure 4b). To confirm that JNK kinase is responsible for TAp73 phosphorylation, we pretreated the cells with JNK-specific inhibitor SP600125, which ablated the phosphorylation of threonine residues in TAp73 (Figure 4d). Interestingly, WN, an analog of WA and PI (Supplementary Figure S5A), induced TAp73 protein levels but did not promote the phosphorylation of TAp73 (Figure 4d, lanes 5 and 6). Additionally, JNK inhibitor not only impeded the phosphorylation of TAp73 but also prevented WA-induced growth inhibition (Figure 4e, Supplementary Figure S7B). In contrast, an inhibitor of p38 kinase (SB203580) did not prevent the growth inhibition triggered by WA (Figure 4f, Supplementary Figure S7C), thus our findings imply that JNK kinase is responsible for TAp73 phosphorylation and mediates the anti-proliferative activity of WA.

### ROS-activated JNK stabilizes TAp73 protein levels via NRF2–NQO1 axis, which is synthetic lethal with the activation of TAp73 transcriptional activity

**Figure 5:**
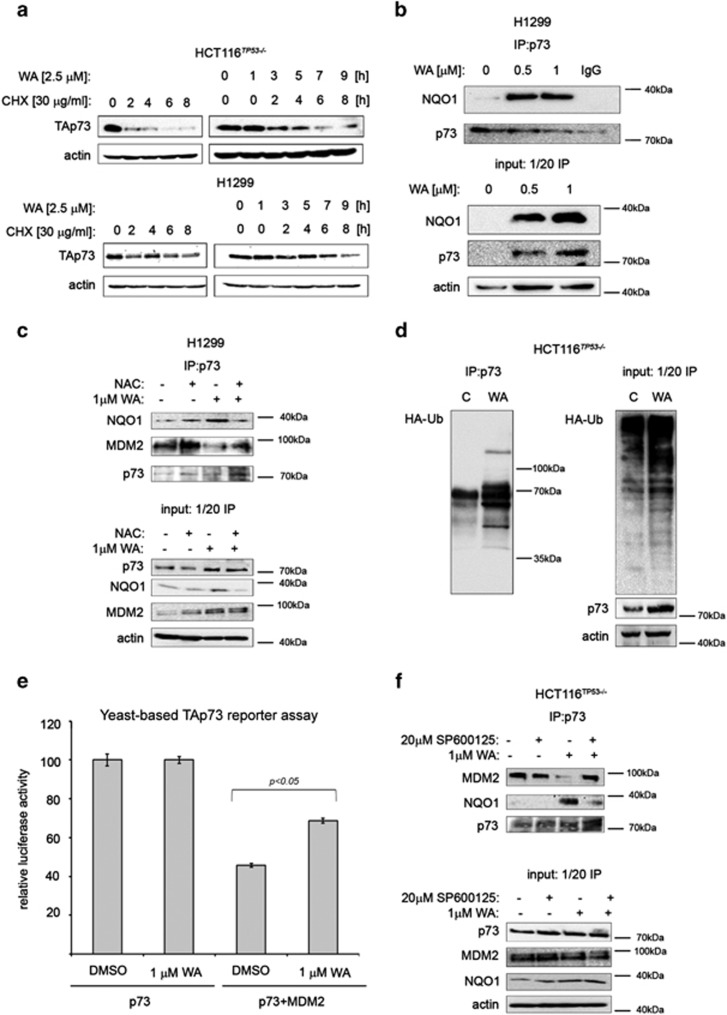
JNK stabilizes TAp73 by Nrf2–NQO1 axis, which is synthetic lethal with TAp73 phosphorylation. (**a**) TAp73 is stabilized in HCT116^*TP53*−/−^ and H1299 cells pretreated for 1 h with WA as shown by CHX chase experiment. (**b**) WA facilitates TAp73/NQO1 binding as revealed by co-immunoprecipitation experiment. (**c**) NAC prevents the binding of NQO1 to TAp73 promoted by WA and prevents WA-mediated inhibition of MDM2-p73 complex. (**d**) Accumulation of Ub-tagged TAp73 in WA-treated HCT116^*TP53*−/−^ cells. (**e**) Co-expression of MDM2 inhibits TAp73-dependent transactivation in a yeast-based reporter assay, which is partially restored by WA (*n*=4). (**f**) In the presence of JNK inhibitor, WA did not inhibit TAp73/MDM2 complex

Next, we focused on elucidating the mechanism of TAp73 protein stabilization upon 1 *μ*M WA as evident in chase experiments (Figure 5a). In the present study, we have found that WA induced oxidative stress and activated the NRF2-related anti-oxidant response (Figures 2a and c), which is prevented by NAC pretreatment. It has been reported previously that ROS-activated JNK phosphorylates NRF2 and promotes its transcriptional activity.^54,55^ This is in agreement with our observation that 1 *μ*M WA stabilizes NRF2 protein as evidenced by the elevated mRNA and protein levels of NF-E2-related transcription factor (NRF2) target genes *NQO1* and *HMOX-1* (Figures 2c and d). NQO1 is a 20S proteasomal gatekeeper, which under oxidative stress directly binds to p53 and TAp73*α* and rescues them from degradation by inhibition of 20S proteolytic activity.^20^ Our immunoprecipitation analysis revealed that WA promoted NQO1 binding to TAp73 (Figure 5b). This binding was at least partially dependent on ROS, as NAC pretreatment significantly reduced the binding of NQO1 to TAp73 upon WA treatment (Figure 5c). Further, NQO1-TAp73 binding promoted accumulation of Ub-tagged TAp73 (Figure 5d), a mark of inhibited proteasomes. As WA directly inhibits catalytic activity of 20S proteasome,^26^ we concluded that both ROS-Nrf2-NQO1 and direct inhibition of 20S contributes to potent TAp73 stabilization by WA.

Phosphorylation of p53 at serine 15 and 20 is a mark of p53 activation leading to ablation of p53/MDM2 complex, p53 protein stabilization and promotion of the binding of p53 co-activators.^56^ As TAp73 transcriptional activity is abrogated by overexpressed MDM2 and MDMX proteins, which directly bind to and inhibit transactivation domain of TAp73,^52^ we investigated whether WA treatment could ablate the interaction between TAp73 and MDM2. To address this, we first used a defined yeast-based reporter system, which detects TAp73 transcriptional activity by using as a readout the functionality of a TAp73-dependent luciferase reporter. As MDM2 does not degrade TAp73, thus in this setup the inhibition of reporter is related to the direct binding of MDM2 to TAp73.^57^ Co-transfection of TAp73 with MDM2 inhibited the TAp73 reporter, which was counteracted by WA treatment (Figure 5e). In addition, WA efficiently disrupted TAp73/MDM2 complex in HCT 116^*TP53*−/−^ cells (Figure 5f), which was dependent on ROS as manifested by the lack of inhibition in NAC pretreated samples (Figure 5c).

To elucidate whether the TAp73 phosphorylation is relevant for the ablation of TAp73/MDM2 complex, we pretreated the cells with JNK inhibitor prior to WA treatment. We found that inhibition of JNK kinase prevents the release of TAp73 from the inhibitory complex with MDM2 protein (Figure 5f).

Hence, we concluded that WA-induced oxidative stress promotes JNK-dependent TAp73 phosphorylation, which leads to the inhibition of TAp73/MDM2 complex and TAp73 activation.

Next, we strived to investigate whether other inhibitors of 20S proteasome work in the way similar to WA. To address this, we used its structural analog – WN (Supplementary Figure S5A), which has been reported to inhibit 20S proteasome catalytic subunit.^24^ Surprisingly, we found that WN induced TAp73 at the protein levels but did not promote TAp73 phosphorylation (Figure 4d). Additionally, WN only slightly inhibited proliferation of cancer cells at a high dose (Supplementary Figure S5B) and did not promote apoptotic phenotype in treated cancer cells H1299 and HCT 116^*TP53*−/−^ (Supplementary Figure S5D). We found that this is attributed to the fact that WN does not induce efficient oxidative stress in cancer cells at the concentrations tested (Supplementary Figure S4D).

Taken together, our data suggests that WA by inducing ROS activates JNK-dependent phosphorylation and consequent activation of TAp73 by targeting TAp73/MDM2 interactions. Concurrently, WA promotes NQO1-driven stabilization of TAp73, which confers synthetic lethality with JNK-related TAp73 activation in cancer cells.

## Discussion

TAp73 is a tumor suppressor that together with TAp63 can recognize many of p53 target genes involved in cell cycle regulation and apoptosis.^50,58,59^ In addition, TAp73 has also unique roles in neuronal stem cells' differentiation^60^ and metabolism.^37^ However, the critical function of TAp73 in tumor suppression is still not fully resolved due to the tissue-specific response deriving from the existence of the p73 isoforms often possessing opposing functions to each other.^33^ This makes the estimation of TAp73 clinical relevance much more complex when compared with p53.^33^ Unlike *TP53*, *TP73* gene is rarely mutated, and the functionality of TAp73 is mainly ablated by inhibitory interactions with ΔNp73, MDM2, MDMX, iASPP or mutant p53.^52^ Thus, targeting protein–protein interactions or modulating pathways promoting TAp73 posttranslational modifications serve as the promising approach for treatment of tumors where p53 is lost or mutated. Several small molecules have recently been reported to positively affect TAp73 apoptotic pathway, including RETRA,^44^ Nutlin-3^42^ and rapamycin;^61^ however, their widespread application to promote p73-dependent tumor regression is still limited.^34^

Eliciting the mechanism underlying cell death evoked by pharmacologically activated TAp73 is of great relevance for the future effective utilization of TAp73 functionality to treat tumors.^40^

In the present work, we report that non-genotoxic oxidative stress synergizes with the inhibition of 20S proteasome to effectively trigger TAp73-related apoptosis in cancer cells using single agent – small-molecule WA. We have shown that WA triggers oxidative stress and that the ROS-activated JNK kinase promotes potent induction of TAp73 protein levels. Further, we elucidated the mechanisms downstream of JNK, which has a crucial role in TAp73 activation in cancer cells where *TP*53 gene is lost or depleted.

Previously, Selivanova and colleaques have shown that the inhibition of thioredoxin reducatase (TrxR), important anti-oxidant enzyme often upregulated in cancers, is synthetic lethal with p53 activation and that ROS-activated JNK mediates this effect.^46^ Here, using two small-molecule inhibitors of the 20S proteasome, we demonstrated that the activation of JNK and inhibition of 20S proteasome confers synthetic lethality in cancer cells.

**Figure 6:**
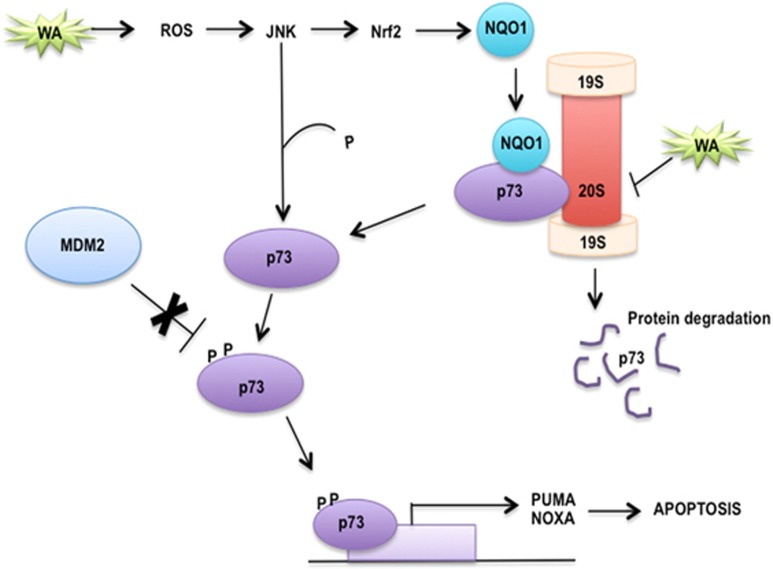
Model showing synergistic mechanism of TAp73 activation by WA via JNK, which is synthetic lethal with inhibition of TAp73 proteasomal degradation. ROS insult activates JNK, which triggers NRF2-dependent expression of NQO1. NQO1 binds to TAp73 and prevents its degradation by 20S proteasome, which leads to TAp73 protein stabilization. JNK also puts phosphorylation marks to promote TAp73 transcriptional activity by ablating TAp73/MDM2 complex. This leads to the activation of proapoptotic PUMA and NOXA, which is indispensable for efficient tumor killing. Thus dual targeting of TAp73/MDM2 and 20S proteasome leads to potent apoptosis in cancer cells deficient for p53

The approval of bortezomib for the treatment of multiple myeloma^21^ and the ongoing clinical trials with the new, potent proteasomal inhibitor AP15 recently identified in a cell-based screen,^21,62^ provide solid rationale for targeting proteasomes for effective anti-cancer treatment. p53 and TAp73 can be stabilized upon IR-mediated ROS in a way independent from ubiquitin, by direct binding to NQO1,^20^ which facilitates the inhibition of 20S proteasome and rescues p53 and TAp73 from degradation. However, the upstream mechanisms leading to the stabilization of TAp73 by ROS insults were not previously described. Our data imply that WA-induced ROS activates JNK kinase, which promotes stabilization of NRF2 transcription factor. This triggers expression of phase II anti-oxidant effectors, including HO-1 and NQO1. Next, we showed that stabilized NQO1 binds to TAp73*α* and promotes accumulation of Ub-tagged TAp73 (Figures 5c and d). This is in agreement with the study by Asher *et al.*,^20^ who demonstrated that NQO1 is critical for Ub-independent turnover of p53 and TAp73. By employing small-molecule WN, an analog of WA and effective inhibitor of 20S proteasome,^25^ we discovered a novel mechanism leading to synthetic lethal effect in cancer cells, converging on ROS-dependent, JNK-driven phosphorylation and activation of TAp73 and simultaneous TAp73 protein stabilization. Based on our data, we propose a model showing how ROS-activated JNK stabilizes TAp73 through activation of NRF2 and its downstream target NQO1, which together with the release of TAp73 from MDM2 leads to activation of TAp73-dependent apoptotic response (Figure 6). We speculate that the activation of the proapoptotic PUMA might further lead to the amplification of ROS insult in cancer cells and provide additional fueling of the activating signals to JNK in a way similar to how we have recently described for the pharmacologically activated p53.^46^

Elucidating the mechanisms by which pharmacologically activated TAp73 promotes apoptosis in cancer cells but not in normal cells is of great importance for the effective therapeutic targeting of TAp73 in the clinical setting. It has been shown that restoration of p53 functionality in advanced tumors but not in early lesions leads to potent cancer regression.^56^ Our present findings imply that WA by promoting ROS provokes accumulation of NQO1, which inhibits 20S proteasome and stabilizes TAp73. Our finding is novel as it describes previously unrecognized pathway of efficient activation of TAp73. We speculate that the same mechanism triggered by a small-molecule WA can lead to the activation of all p53 family members, including p53, and this will be the subject of future studies. Similar mechanism regarding the protein maintenance by activated NQO1 was reported for the translation initiation factor 4GI (eIF4GI); however, in that study, the link to how the oxidative stress might contribute to this process was not recognized.^63^

Thus our novel findings describing synthetic lethality between inhibition of proteasome system and ROS-dependent activation of MAPK JNK kinase for potent cancer cell death constitutes an attractive anti-cancer approach, which might serve as an important rationale for the development of effective treatment of ROS-compromised tumors by small molecules like WA.

## Materials and Methods

### Cell lines and chemicals

Tumor cell lines HCT116^*TP53*−/−^ and RKO^*TP53*−/−^ were provided by B Vogelstein, John Hopkins University, USA. HCT116^*TP53*−/−^, RKO^*TP53*−/−^ H1299 and NHDFs were maintained in Iscove's Modified Dulbecco's Medium (IMDM) supplemented with 10% fetal bovine serum (FBS), L-glutamine (2 mM) and penicillin/streptomycin (10 units/ml). All cell lines were cultured at 37 °C in a humidified incubator with 5% CO_2_.

WA was purchased from Sigma-Aldrich (Santa Cruz, St. Louis, MO, USA) and dissolved in 100% DMSO to 1 mM concentration. Cisplatin (CDDP) was purchased from Sigma-Aldrich and diluted in 0.9% NaCl to 25 mM solution and added to the culture medium of 25 *μ*M final concentration. NAC (Sigma-Aldrich) was dissolved in dH_2_O to a 0.5 M solution and further added to cell culture medium to a 5 mM concentration. SP600125 JNK inhibitor was purchased from Sigma-Aldrich, dissolved in 100% DMSO and used at 20–30 *μ*M concentration.

zVAD-FMK was purchased from Sigma-Aldrich and used at 10 *μ*M concentration.

### siRNA and plasmids

For TAp73 knockdown, following siRNAs were used: TAp73_318 sense: 5′-AGGGCAUGACUACAUCUGU-3′ antisense: 5′-ACAGAUGUAGUCAUGCCCU-3′ and TAp73_223 sense: 5′-ACCAGACAGCACCUACUUC-3′ antisense: 5′-GAAGUAGGUGCUGUCUGGU-3′. Transfection with siRNA (10 nM) was performed with HiPerfect Qiagene (Qiagen, Germantown, MD, USA) reagent for 24 h (for WB analysis) or 48 h (for colony-formation assay) following the manufacturer's instruction.

Plasmid encoding for TAp73*α* isoform was a kind gift from Dr. Matthias Dobbelstein. Transfection was performed with Lipofectamine 2000 Reagent (Life Technologies, Thermo Fisher Scientific, Carlsbad, CA, USA), according to the manufacturer's protocol.

### Generation of stable TAp73 knockdown cell lines

To establish stable TAp73 knockdown HCT116*^TP53−/−^* and H1299 cell lines, we used two different shRNA constructs directed against human TAp73. The shRNA oligos were cloned into pLKO.1-puro vector (Addgene, Cambridge, MA, USA; plasmid 8453) as previously described by Stewart *et al.*;^64^ oligo sequences were 5′-CCGGACCAGACAGCACCTACTTCTTCAAGAGAGAAGTAGGTGC TGTCTGGTTTTTTG-3′ and 5′-CCGGGAACGGATTCCAGCATGGATTCAAGAGATCCA TGCTGGAATCCGTTCTTTTTG-3′. Packaging and envelope constructs pCMVΔ8.2 and pMD.G-VSV-G were used for lentivirus generation via transfection into HEK293T cells as previously described in Szulc *et al.*^65^ In all, 16 000 cells/well were seeded in 96-well plates and transduced with either control lentivirus or lentivirus containing TAp73 shRNA and selected by growing in puromycin (1 *μ*g/ml) for 3–4 weeks.^64,65^

### Anchorage-independent growth by soft agar assay

1.2% Low-melting temperature agarose (Sea plaque agarose, Lonza Group Ltd, Basel, Switzerland) was melted and mixed with an equal volume of warm 2 × Dulbecco's Modified Eagle's Medium (DMEM) (containing 20% FBS, 4 mM L-glutamine, 2% penicillin/streptomycin) to prepare 0.6% base agarose. A total of 600 *μ*l of this mixture was added per 12-well and solidified for 30 min at RT. The top agarose (0.4% agarose) was prepared mixing 0.8% low-melting temperature agarose with 2 × DMEM-containing cells. Ten thousand cells per 12-well was added onto the base agarose and solidified for 30 min at RT. In all, 500 *μ*l medium containing WA was added onto top agarose. After 1 week of culture, colonies were stained with MTT solution (10 mg/ml) and scanned.

### Primary MEFs

TAp73^+/+^ and TAp73^−/−^ primary MEFs were isolated from E13.5 embryos and cultured in DMEM supplemented with 10% FBS, 2 mM L-glutamine and 55 *μ*M b-mercaptoethanol. All animal experiments were conducted in accordance with the guidelines of Karolinska Institute and approved by the Stockholm's North Ethical Committee of Animal Research.

### Cell viability assay

For viability studies, cells were plated at a concentration of 5 × 10^3^ cells/well, and WA was added in IMDM medium at increasing concentrations, and 25 *μ*M CDDP was used as a positive control. After 48 h, absorbance of WST-1 (2-(4-iodophenyl)-3-(4-nitrophenyl)-5-(2,4–disulfophenyl)-2H-tetrazolium, monosodium salt, Roche Diagnostics, Penzberg, Germany) dye was measured in a Perkin-Elmer (Waltham, MA, USA) microplate reader, and cell viability was calculated.

### Clonogenic survival assay

Cells were plated at concentrations of 1.5 × 10^5^ per well or 2 × 10^5^ for stable TAp73 knockdown cell lines, and WA was added in increasing concentrations. After 48 h, medium was removed, and cells were washed with PBS, followed by 70% ethanol and stained with 0.5% crystal violet solution in PBS. Densitometric analysis of relative well density was performed using the ImageJ software (Bethesda, MD, USA).

### Quantitative PCR

mRNA was isolated and reverse transcribed to cDNA according to the manufacturer's instructions (Bio-Rad, Sundbyberg, Sweden). For qPCR, the following concentrations were used: 150 nM; 10 ng cDNA; 7.5 *μ*l 2 × master mix (Bio-Rad); water to a total of 15 *μ*l; primers used: *NOXA* (PMAIP1) forward 5′-AAGTGCAAGTAGCTGGAAG-3′, reverse: 5′-TGTCTCCAAATCTCCTGAGT-3′, *PUMA* forward: 5′-CTCAACGCACAGTACGAG-3′ and reverse: 5′-GTCCCATGAGATTGTACAG-3′, *NFLE2E* forward: 5′-TCCCAGCAGGACATGGATTT-3′ and reverse: 5′-GCTCATACTCTTTCCGTCGC-3′, *HMOX-1* forward: 5′-TTCACCTTCCCCAACATTGC-3′ and reverse: 5′-TATCACCCTCTGCCTGACTG-3′, *Bcl-2* forward: 5′-GCCTTCTTTGAGTTCGGT-3′ and reverse: 5′-AGTTCCACAAAGGCATCC-3′, *Bid* forward: 5′-GTGAGGTCAACAACGGTTCC-3′ and reverse: 5′-TGCCTCTATTCTTCCCAAGC-3′, *Bim* forward: 5′-TGGCAAAGCAACCTTCTGATG-3′ and reverse: 5′-GCAGGCTGCAATTGTCTACCT-3′, *ΔNp73* forward: 5′-CAAACGGCCCGCATGTTCCC-3′ and reverse: 5′-TTGAACTGGGCCGTGGCGAG-3′, and *TAp73* forward: 5′-GGGAATAATGAGGTGGTGGG-3′ and reverse: 5′-AGATTGAACTGGGCCATGAC-3′.

### ROS measurement

ROS were measured as described previously.^66^ Briefly, cells were treated with WA or pretreated with 5 mM NAC or 100 *μ*M H_2_O_2_ for 6 h and stained with 10 *μ*M DCF-DA (Sigma-Aldrich) in serum-free IMDM for 45 min. After three washing steps with PBS, cells were trypsynized and suspended in PBS. Samples were analyzed by flow cytometry, acquiring 10 000 cells per sample. Data analysis was performed with the CELLQuest software (CELLQuest, Franklin Lakes, NJ, USA).

### Cell cycle analysis by flow cytometry

Cells were stained with propidium iodide solution (Sigma-Aldrich). Samples were washed, suspended in PBS and analyzed by flow cytometry, covering acquisition of 10 000 cells per sample. Data analysis was performed with the CELLQuest software.

### Co-immunoprecipitation and western blottig analysis

Cells for both whole-cell lysates and immunoprecipitates were solubilized in lysis buffer: 25 mM Tris HCl, pH 8.0, 150 mM NaCl, and 1% Nonidet P-40 (0.5% for co-IP). For co-IP, 1 mg protein was precleared with 10 *μ*l Dynabeads Protein A (Invitrogen, Carlsbad, CA, USA) coupled with normal rabbit IgG. Precleared lysates were then immunoprecipitated for 16 h with 1.5 *μ*g of *α*-TAp73 rabbit polyclonal antibody (Bethyl Laboratories, Montgomery, TX, USA). Immunocomplexes were incubated with 20 *μ*l of Dynabeads Protein A for 5 h at 4 °C to form complexes. The immunoprecipitates were washed two times with 300 *μ*l of PBS and two times with 200 *μ*l of lysis buffer, resuspended in 15 *μ*l of lysis buffer and 10 *μ*l of 6 × Laemmlibuffer and boiled prior to western blotting analysis. The antibodies used for detection were: anti-p73 antibodies (Bethyl Laboratories), anti-MDM2 (Santa Cruz Biotechnology, Dallas, TX, USA), anti-NQO1 (Santa Cruz Biotechnology), anti-HA (Santa Cruz Biotechnology) and anti-phospho-threonine (Cell Signaling, Danvers, MA, USA).

Western blotting was performed according to the standard protocol. Briefly, 100 *μ*g of total cell lysate (200 *μ*g for phosphorylated protein detection) was subjected to electrophoresis, and the following antibodies were used to detect proteins: anti-TAp73 (Bethyl Laboratories), anti-BAX (Santa Cruz Biotechnology) anti-PUMA (Cell Signaling), anti-HO (Santa Cruz Biotechnology), anti-NRF2 (Santa Cruz Biotechnology), anti-Pp73(Tyr99), anti-pJNK (Cell Signaling) anti-pp38 (Cell Signaling), anti-Bid (Santa Cruz Biotechnology), and anti-actin (Sigma).

### Cycloheximide (CHX) protein stability assay

For CHX chase experiment, H1299 and HCT116^*TP53*−/−^ cells were pretreated for 1 h with WA. In all, 30 *μ*g/ml CHX was then added to block protein translation. Cell pellets were collected, solubilized in lysis buffer and analyzed by western blotting as previously described.

### Caspase activation assay

Detection of caspases was measured after 16 h treatment with WA with the use of FLICA (Fluorescent-Labeled Inhibitor of Caspases) apoptosis and caspase detection kit (ImmunoChemistry Technologies, Bloomington, MN, USA). In brief, cells were collected, washed with PBS and stained according to the manufacturer's protocol. Caspase activity was measured by flow cytometry, and collected results were analyzed with CELLQuest software.

### Yeast-based reporter system

The TAp73-dependent yeast reporter strain yLFM-PUMA containing the luciferase cDNA cloned at the *ADE2* locus and expressed under the control of a promoter element derived from the human PUMA gene was transfected with pTSG-p73 and pRB-MDM2 (generously provided by Dr. R Brachmann, University of California, Irvine, CA, USA) and selected on double drop-out media for TRP1 and HIS3. Expression of p73 was controlled by the addition of galactose in the culture medium, while MDM2 expression was constitutive. Luciferase activity was measured 16 h after the shift of double transformant cells to galactose-containing selective media and the addition of WA or DMSO. Presented are average relative light units and the S.E. obtained from three independent experiments each containing four biological repeats. In all cases *t* Student test was performed to assess statistically relevant differences.

### Comet assay

DNA strand breaks were detected by comet assay as previously described.^67^ Briefly, following treatment, cells were mixed with 100 *μ*l of 1% low melting agarose (Prona, Reducta LM, Poland), and 75 *μ*l of this cell–agarose mixture was spread on microscopic slides precoated with 1% agarose. A third layer of 0.5% low-melting agarose (75 *μ*l) was applied over the layer of agarose with the cell suspension. Slides were incubated for 1 h in a lysis solution. The microscopic slides were immersed in an alkaline buffer (300 mM NaOH, 1 mM disodium EDTA) and subjected to electrophoresis at 1 V/cm for 30 min. Stained cells were analyzed under a fluorescence microscope (Nikon, Tokyo, Japan PCM-2000) using the Cometscore software (Tri Tek Corp., Sumerduck, VA, USA). Images of 20 cells from three slides were analyzed. Densities were measured for each image in two areas: the whole cellular DNA and the area containing only the comet head. Results are presented as tail moment, which is the percentage of DNA in the comet tail multiplied by the tail length.

## Abbreviations

ROS: reactive oxygen species
PI: proteasome inhibitor
WA: Withaferin A
MAPK: mitogen-activated protein kinase
UPP: ubiquitin proteasome pathway
IP: immunoprecipitation
Nrf2: NF-E2-related transcription factor
NAC: N-acetyl-L-cysteine
NQO1: NADPH quinone oxidoreductase
NAC: *N*-acetyl-L-cysteine
JNK: Jun N-terminal kinase
NHDF: normal human diploid fibroblasts
MEF: mouse embryonic fibroblasts
HMOX-1: heme oxygenase 1
iASPP: inhibitor of apoptosis-stimulating protein of p53
