# Correction to: JNK–NQO1 axis drives TAp73-mediated tumor suppression upon oxidative and proteasomal stress

**DOI:** 10.1038/s41419-018-0654-2

**Published:** 2018-06-07

**Authors:** A. Kostecka, A. Sznarkowska, K. Meller, P. Acedo, Y. Shi, H. A. Mohammad Sakil, A. Kawiak, M. Lion, A. Królicka, M. Wilhelm, A. Inga, J. Zawacka-Pankau

**Affiliations:** 1 Department of Biotechnology, Intercollegiate Faculty of Biotechnology, University of Gdansk and Medical University of Gdansk, Gdansk, Poland; 2 Department of Microbiology, Tumor and Cell Biology, Karolinska Institutet, Stockholm, Sweden; 3 Centre for Integrative Biology, CIBIO, University of Trento, Mattarello, Italy

**Correction to**: *Cell Death Dis.* **5**, e1484 (2014); 10.1038/cddis.2014.408; published online 23 October 2014.

Following publication of their article JNK-NQO1 axis drives TAp73-mediated tumor suppression upon oxidative and proteasomal stress. *Cell Death Dis.* 2014, 5:e1484, the authors noted a mistake in Fig. 2b, in that, the wells of the crystal violet plates showing growth inhibition induced by withaferin A in HCT 116^*TP53−/−*^ cells were erroneously duplicated for 1 and 2.5 μM WA. The correct wells for 2.5 μM WA are now included in new Fig. 2b.

The authors apologise for any inconvenience caused.

**Fig. 2:**
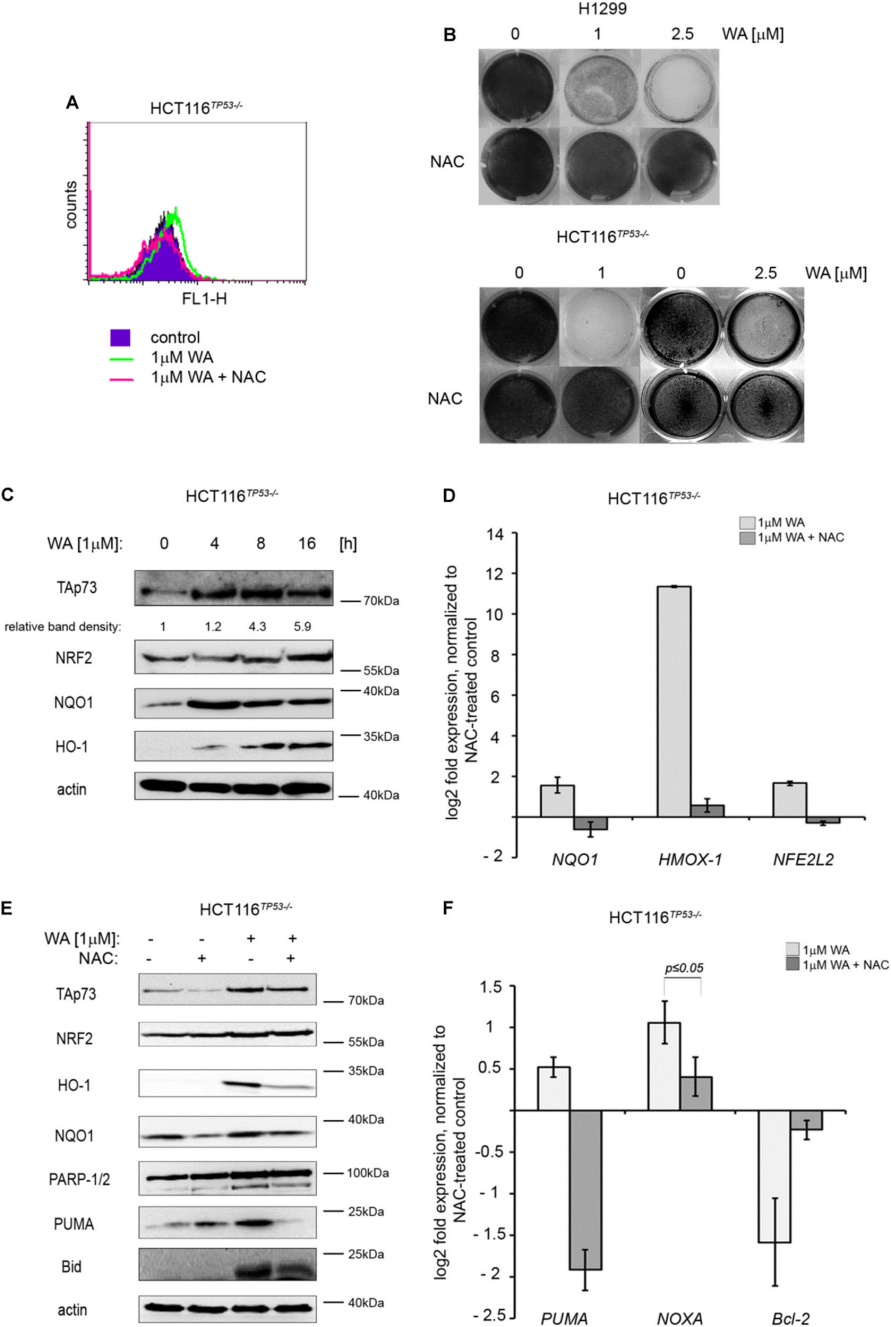
.

