# Supplementary material for: JNK–NQO1 axis drives TAp73-mediated tumor suppression upon oxidative and proteasomal stress
Source: Cell Death Dis. 2014 Oct 23;5(10):e1484–. doi: 10.1038/cddis.2014.408 (PMC4649515; doi:10.1038/cddis.2014.408)

Supplementary Figure 1

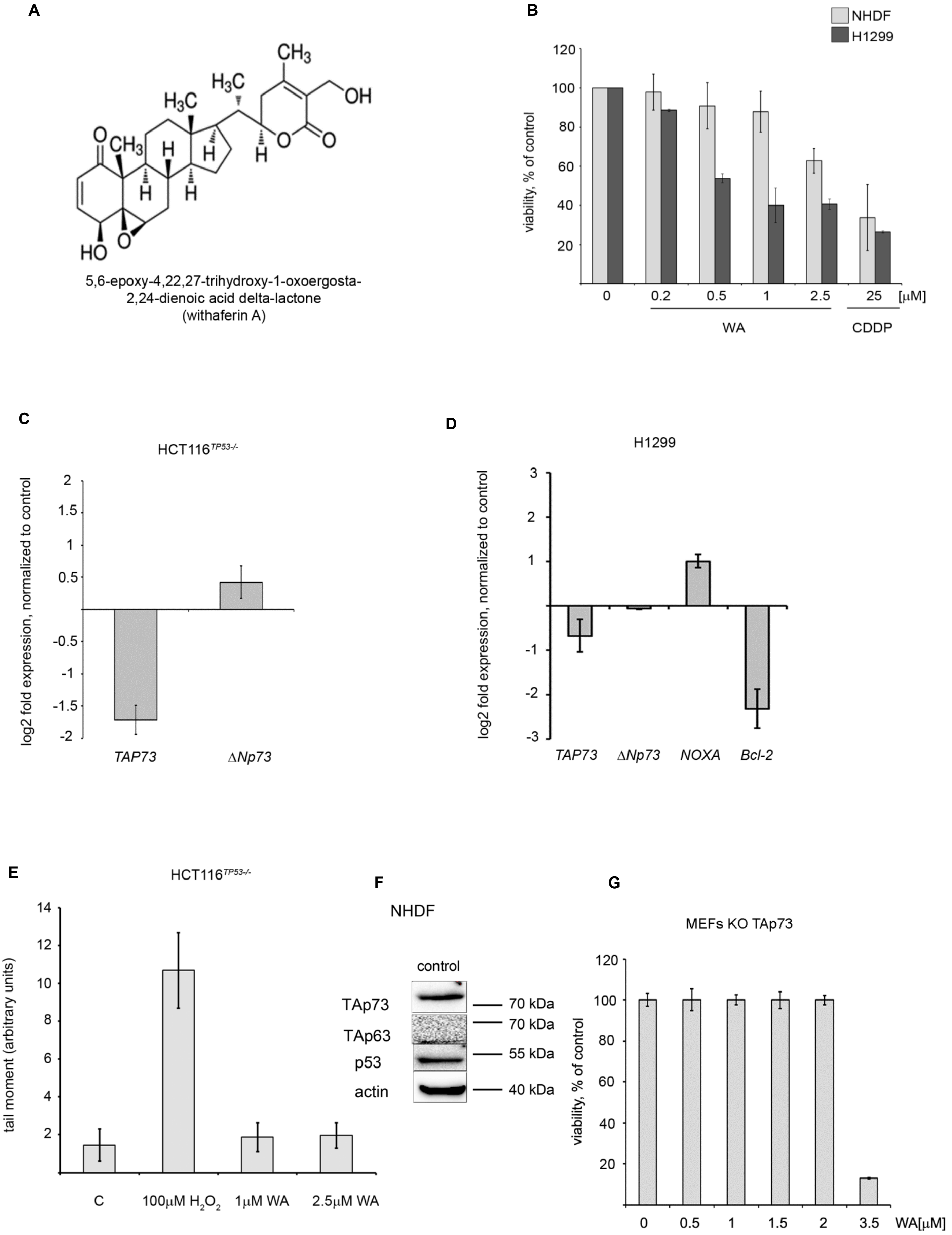

Supplementary Figure 2

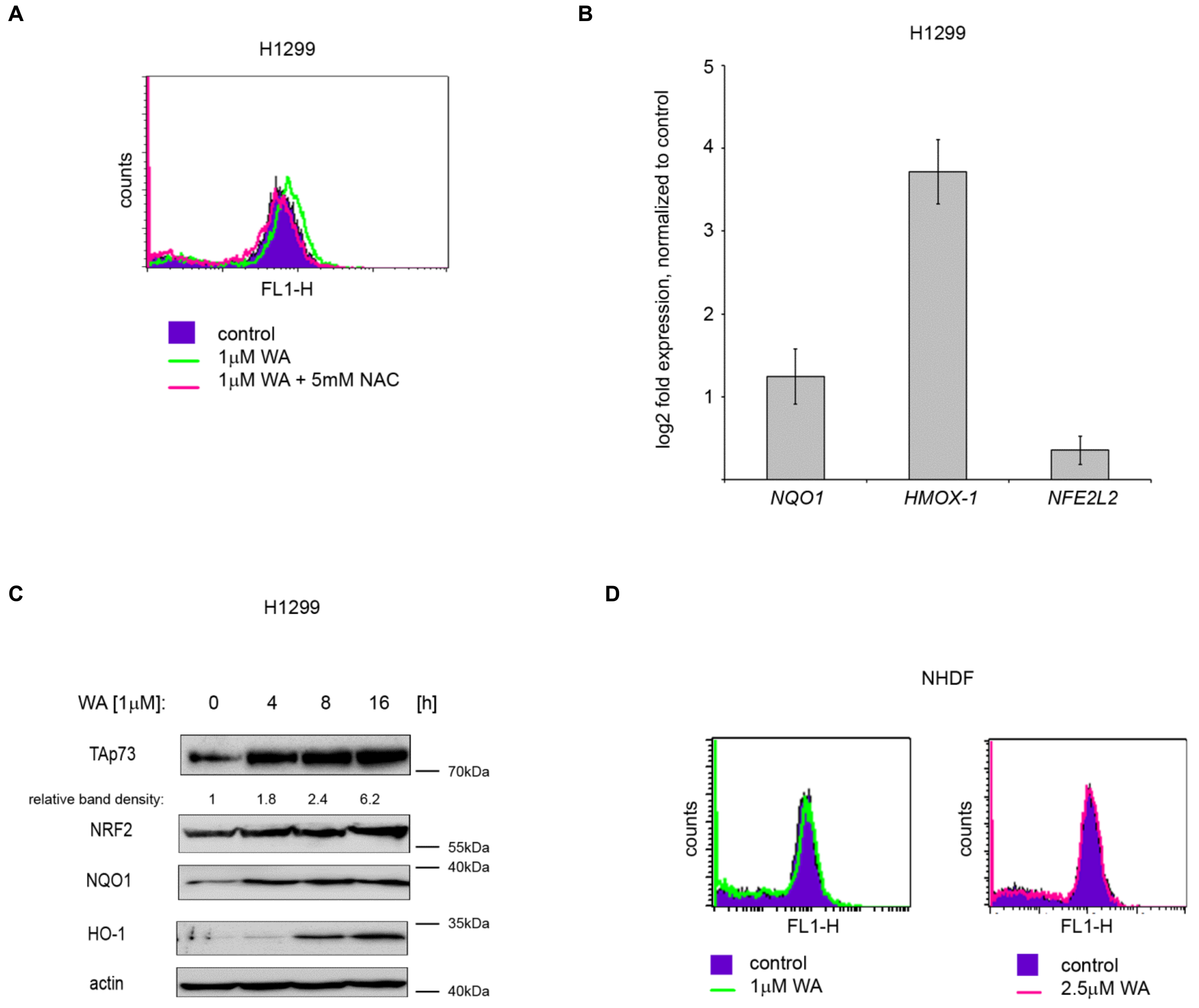

Supplementary Figure 3

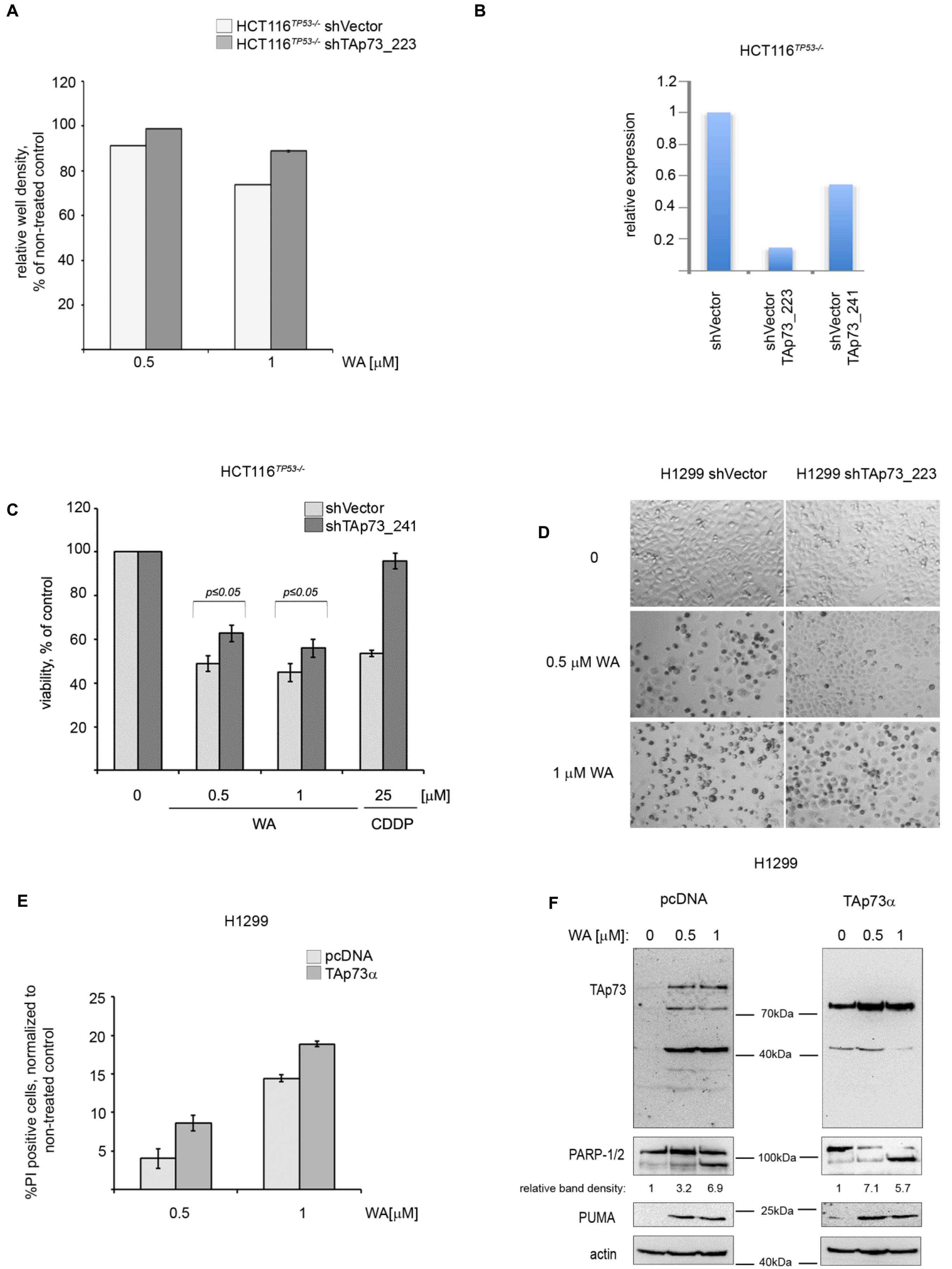

Supplementary Figure 4

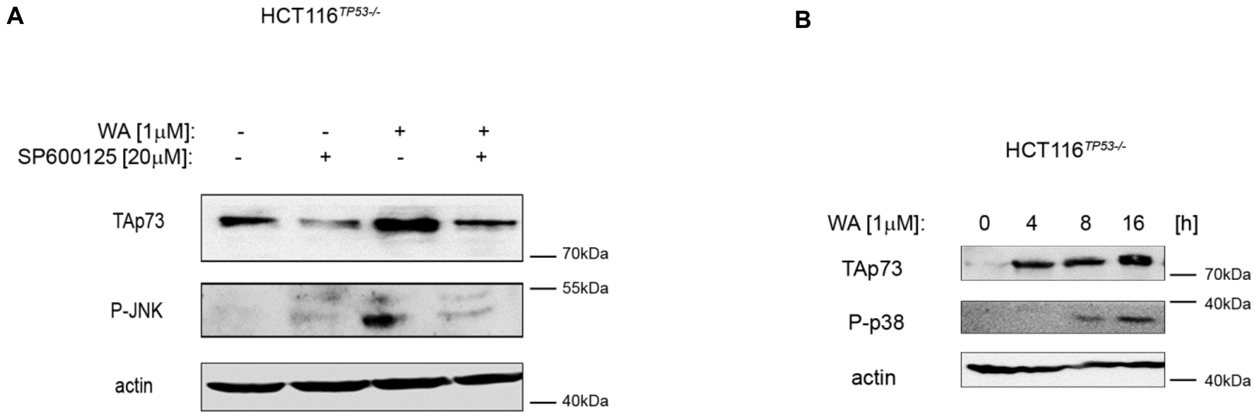

Supplementary Figure 5

A

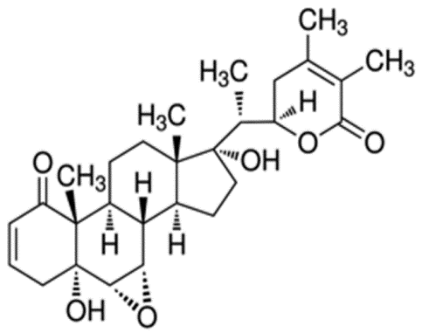

(5a,6a,7a,22R)-6,7-Epoxy-5,17,22-trihydroxy-1-oxoergosta-2,24-dien-26-oic acid d-lactone (withanone)

B

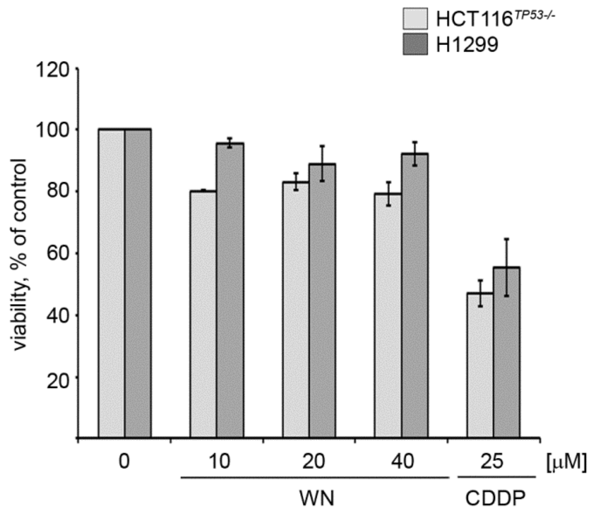

C

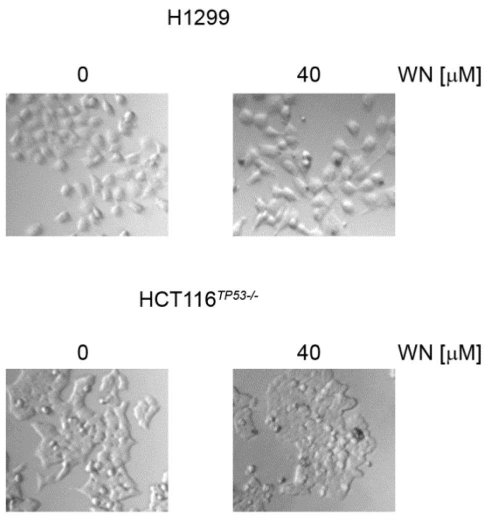

D

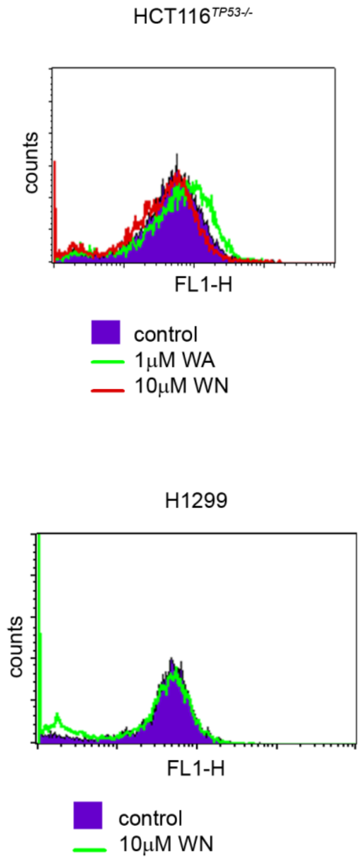

Supplementary Figure 6

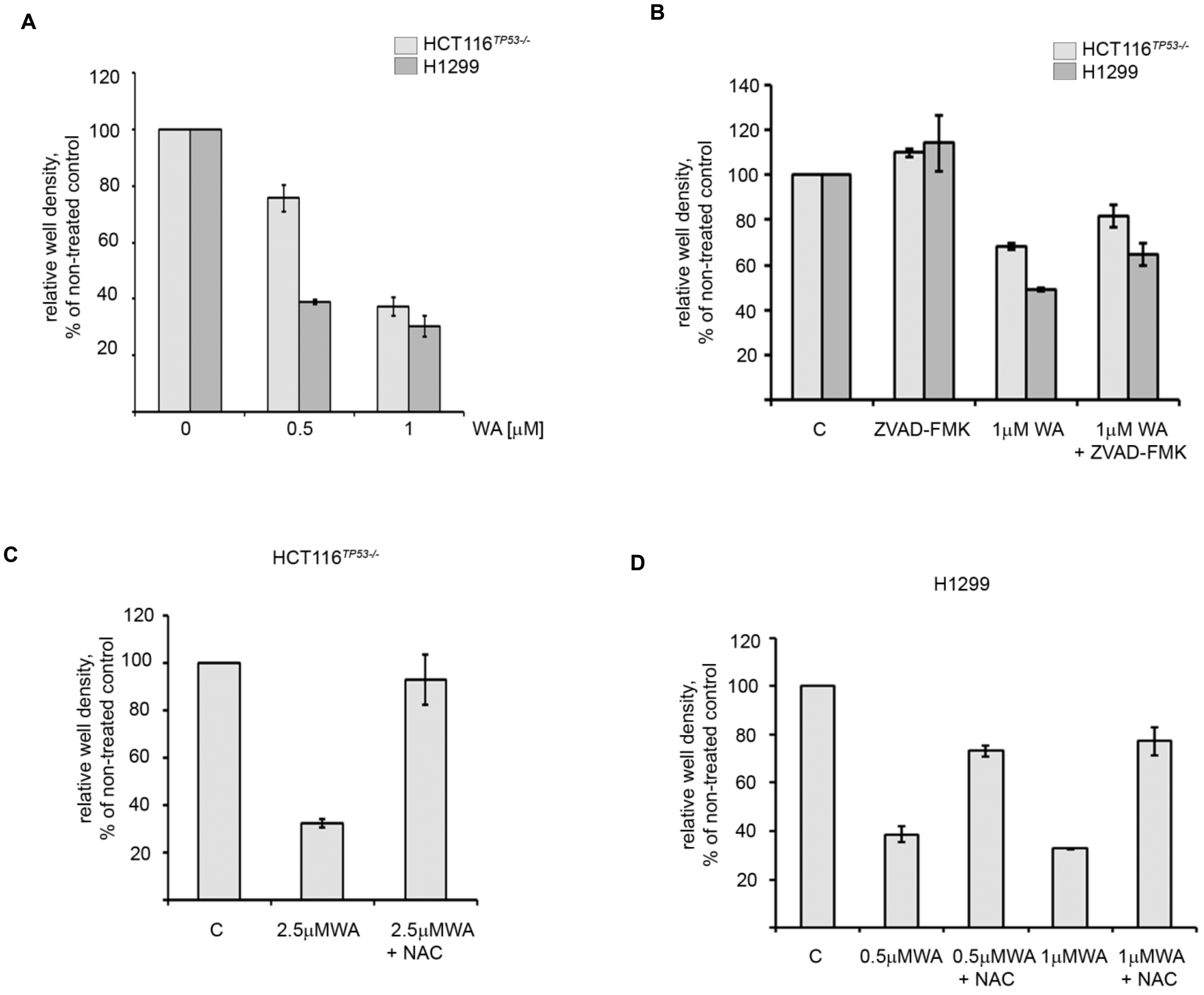

Supplementary Figure 7

A

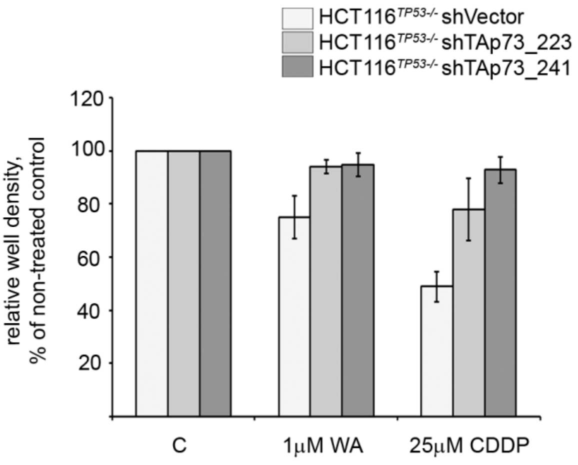

B

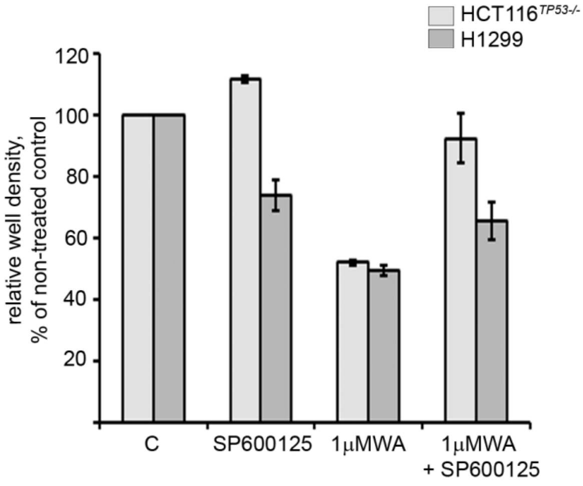

C

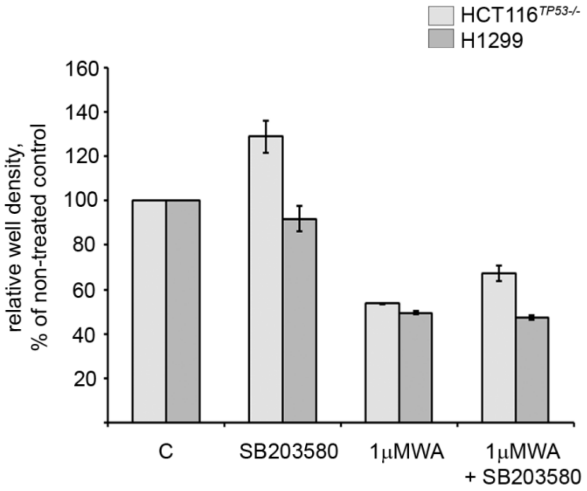

Supplement: Supplementary Figures [file cddis2014408x1.pdf]
