# Supplementary material for: JNK–NQO1 axis drives TAp73-mediated tumor suppression upon oxidative and proteasomal stress
Source: Cell Death Dis. 2014 Oct 23;5(10):e1484–. doi: 10.1038/cddis.2014.408 (PMC4649515; doi:10.1038/cddis.2014.408)
Supplement: Supplementary Figure Legends [file cddis2014408x2.doc]

**Supplemental Figure Legends**

**Figure S1. WA inhibits proliferation and triggers apoptosis in p53 deficient cancer cells.**

(A) Chemical structure of withaferin A (WA). (B) WA inhibits viability of H1299 cancer cells but has little impact on viability of normal human diploid fibroblasts (NHDF) at the concentrations tested (n=3). (C) WA does not affect *TAP73* and *ΔNp73* expression levels in HCT116*^TP53-/-^* cells as presented by qPCR. (D) WA promotes expression of *NOXA* and downregulates expression of anti-aopoptotic *BCL*2 in H1299 cells but does not affect *TAP73* and *ΔNp73* expression levels, as revealed by qPCR. (E) Quantification of comet assay indicates that WA does not promote DNA strand breaks, thus is non genotoxic in HCT116*^TP53-/-^* cells. Values are means (±SD) of three independent experiments (n=3). H_2_O_2_ was used as positive control. (F) Immunoblot representing protein levels of TAp73, TAp63 and p53 in NHDF. (G) Toxicity of WA in MEFs knock-out for TAp73.

**Figure S2. WA triggers oxidative stress response.**

(A) Cytometric analysis of DCF-DA stained H1299 cells showing ROS induction by WA. (B) Quantitative gene expression analysis of WA-treated H1299 reveals significant induction of *NQO1* and *HMOX-1* genes. (C) WA induces NRF2 and its downstream anti-oxidant effectors NQO1 and HO-1 at the protein level. (D) ROS is not induced in NHDF by WA.

**Figure S3. TAp73 is important for WA anti-tumor activity.**

A) Anchorage-independent growth soft agar assay presenting decreased colony formation upon stable TAp73 knock down in HCT116*^TP53-/-^* cells after WA treatment. (B) qPCR showing the efficiency of stable knock-down of *TP*73 using 2 different shRNAs. (C) Viability test showing that p73 depletion de-sensitizes cells to WA used at IC50 concentration. Statistical analysis performed using Student’s *t* test (D) Microscopic analysis indicates less efficient promotion of apoptotic phenotype by WA after 24h upon p73 depletion in H1299 cells. (E) Propidium iodide-stained cells show increased sensitivity to WA upon TAp73α overexpression. (F) Ectopic expression of TAp73 promotes more potent induction of pro-apoptotic PUMA and PARP cleavage upon WA treatment.

**Figure S4. Withaferin A promotes JNK-dependent accumulation of TAp73 in cancer cells.**

(A) Immunoblot performed in HCT116*^TP53-/-^*  pre-treated with JNK inhibitor (SP600125), followed by 1µM WA treatment, representing protein levels of TAp73 and phosphorylated JNK. (B) WA triggers phosphorylation of p38 kinase in HCT116*^TP53-/-^* cells.

**Figure S5. The WA analog, withanone does not exert anti-cancer properties due to insufficient ROS induction.**

(A) Chemical structure of withanone (WN). (B) WST-1 proliferation assay in withanone-treated H1299 and HCT116*^TP53-/-^* cells shows little on cell viability. (C) Withanone does not promote apoptotic phenotype in cancer cells even at very high doses. (D) Withanone does not induce ROS.

**Figure S6. Densitometric analysis of clonogenic assay.**

(A) Graph representing densitometric analysis of relative well density of HCT116*^TP53-/-^* and H1299 cells upon WA treatment. (B) Graph representing densitometric analysis of relative well density of HCT116*^TP53-/-^* and H1299 treated with WA and pre-treated with ZVAD-fmk. (C,D) Graphs representing densitometric analysis of relative well density of HCT116*^TP53-/-^* and H1299 treated with WA and pre-treated with NAC.

**Figure S7. Densitometric analysis of clonogenic assay.**

(A) Graph representing densitometric analysis of relative well density of HCT116*^TP53-/-^* stable TAp73 knock-down cells upon WA treatment. (B) Graph representing densitometric analysis of relative well density of HCT116*^TP53-/-^* and H1299 treated with WA and pre-treated with JNK inhibitor (SP600125). (C) Graph representing densitometric analysis of relative well density of HCT116*^TP53-/-^* and H1299 treated with WA and pre-treated with p38 inhibitor (SB203580).
